# Analysis of the Influence of Adhesion on Measured Runway Friction

**DOI:** 10.3390/ma19102073

**Published:** 2026-05-15

**Authors:** Gadel Baimukhametov, Greg White

**Affiliations:** School of Science, Technology and Engineering, University of the Sunshine Coast, Sippy Downs, QLD 4556, Australia; g_b079@student.usc.edu.au

**Keywords:** friction, runway, adhesion, surface tension, asphalt, British pendulum tester, temperature, viscous hydroplaning

## Abstract

Runway friction is a critical factor for aircraft operational safety, yet the role of adhesion in wet friction remains insufficiently understood, especially in areas where tyre rubber contaminates the surface. This study evaluated approximate adhesive contribution for representative common runway surfaces, using contact angle measurements and British pendulum tester friction tests. The results show that approximate adhesion influence varies strongly with surface type: negligible on cement concrete, 16% to 19% on rubber-contaminated asphalt, and up to 49% on roughened rubber. A linear correlation between friction and contact angle confirmed that wetting behaviour governs adhesion-driven friction. Friction tests at different temperatures also confirmed the adhesive nature of the temperature influence on friction. The analysis further indicates that material properties and greater effective surface area correlate with stronger adhesive contributions, explaining material-specific differences in friction performance. These findings may provide a conceptual basis for interpreting variability in continuous friction measurements and suggest the importance of considering adhesion effects in runway surface characterisation and maintenance systems.

## 1. Introduction

Runway friction is an important part of aircraft operational safety. Nowadays, insufficient runway friction is the main contributing factor in up to 20% of runway excursions [1]. For this reason, friction testing is an important part of the runway operation and maintenance. The reliability of friction tests, however, needs improvement. Continuous friction measurement equipment (CFME) is the main tool for runway friction assessment. The International Civil Aviation Organization (ICAO) recommends performing tests at 65 km/h and 95 km/h, which capture friction reduction due to surface degradation and rubber contamination [2]. Different studies, however, report that CFME results change throughout the year due to temperature or humidity [3,4]. To avoid misinterpreting the friction testing results, especially in the cases of heavy rubber contamination, it is important to fully understand the mechanism of friction and the properties of materials affecting friction.

Although different mechanisms of friction and friction loss on a runway have been thoroughly studied [5,6,7], the effect of adhesion on runway friction is still understudied. It is important to understand the role of adhesive friction, especially for contaminated pavement. Consequently, the aim of this research was to evaluate the influence of adhesion on runway friction.

### 1.1. Hysteresis and Adhesive Friction

On a runway, friction is the resisting force between a tyre and the surface that opposes the tyre’s motion. The simplest model describing sliding friction is Amontons’ (or Coulomb’s) law. It can be expressed as shown in Equation (1).

(1) $$F_{fr}=\mu N$$

where *F_fr_* is the friction force, *µ* is the coefficient of friction, and *N* is the normal load. This is a general law and is limited to low loads and the absence of adhesion [8]. Equation (1) also suggests that the friction force is independent of the contact area, which is often not observed in practical experiments.

**Figure 1:**
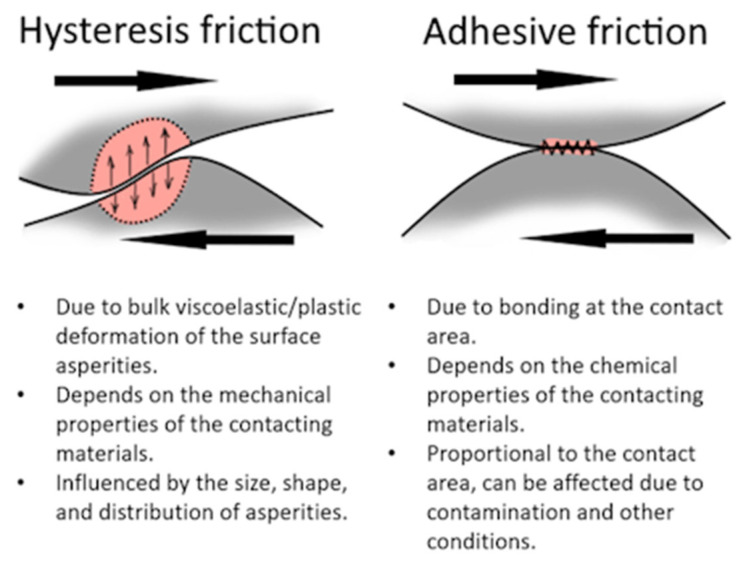
Main mechanisms of friction.

Friction is known to be based on hysteresis and adhesion effects (Figure 1). The adhesion effect is the “sticking” caused by molecular interactions between the two surfaces, while hysteresis is the energy loss due to cyclic deformation during the sliding of one surface over the other. In this case, the contact area can be divided into stick (adhesion) and slip (hysteresis) regions [9]. Although the influence of hysteresis on friction may dominate under certain conditions, adhesive interaction is crucial for the initiation of sliding and enhances surface deformation [9]. The term hysteresis is broad, and it is also important to note that it can be applied to adhesive interactions as well [8,10]. Here, it is applied to the friction effects resulting from the deformation of the sliding tyre and the pavement surface.

During adhesive contact, energy loss, and therefore friction, occurs even for a perfectly elastic impact due to irreversible “snap-on” and “snap-off” effects [11]. The attractive forces between bodies cause energy loss due to a decrease in surface energy. Several theories describe the influence of adhesion on the contact between two bodies. They include the Johnson–Kendall–Roberts–Sperling model [12] and the Derjaguin–Muller–Toporov model [13]. Those two theories are based on the assumption of elastic body interactions. The Maugis–Pollock model builds on the abovementioned models and considers plastic deformation during the interaction [14]. These theories, however, do not account for surface interactions in the presence of contaminants, such as rubber contamination from aircraft tyres.

### 1.2. Wet Friction

Wet friction is more complex because of the friction reduction due to intermolecular interaction between surface and water, as well as water pressure. Squeezing and movement of surfaces cause dynamic and viscous effects. The modelling of dynamic effects is a straightforward problem that can be solved using tools such as finite element modelling (FEM), given the simplicity of the macro-geometry between the interacting surfaces [15,16,17,18]. For the same reason, experimental evaluation of these effects is accurate and reliable [19,20].

The viscous effect requires more complex evaluation due to the strong influence of surface irregularities. Moore’s theory of viscous hydroplaning, which is based on Reynolds’ equation for laminar flow trapped between two moving surfaces, is used today in research and practical applications [21]. According to the two-dimensional form of Equation (2).

(2) $$\frac{\partial }{\partial x_{i}}\left(h_{i}^{3}\frac{\partial p_{i}}{\partial x_{i}}\right)=6\mu U_{0}\frac{\partial h_{i}}{\partial x_{i}}\;$$

where *h_i_* is the distance between two surfaces and a function of *x_i_*, *U*_0_ is the relative velocity between two bodies, *p* is the pressure and *µ* is the viscosity. It is clear that this equation cannot describe the actual contact between bodies, although it can be solved by applying appropriate boundary conditions for pressure or thickness. Due to the laminar flow solution minimal distance between two bodies is limited by the average microtexture roughness [22].

It has been reported that an increase in water temperature reduces the friction during testing [23,24]. However, Equation (2) shows that an increase in temperature should increase friction due to a reduction in viscosity and the subsequent reduction in viscous pressure in the area of contact. That means the mechanism of friction reduction cannot be solely explained by the hysteresis effect and partial viscous hydroplaning.

### 1.3. Disjoining Pressure and Adhesive Friction

Besides viscous and dynamic effects, wet contact is highly affected by the adhesive effects. The key parameter describing the process of contact between two bodies (1 and 2) and a liquid film is the spreading pressure. Spreading pressure *S* describes the behaviour of the line where all three phases meet. When *S* < 0, de-wetting occurs, if *S* > 0 then the liquid film tends to disjoin two surfaces. It can be found using Equation (3).

(3) $$S=\gamma_{1}+\gamma_{2}-\gamma_{12}$$

where *S* is spreading pressure and *γ*_1_, *γ*_2_, and *γ*_12_ are surface energies of the liquid-surface contact and surface-surface contact, respectively.

Another parameter characterising the adhesive contact is the disjoining pressure. The disjoining pressure is a pressure difference in the area of contact caused by intermolecular interactions. The disjoining pressure due to van der Waals forces in a liquid film with thickness *h*_0_ can be found using Equation (4).

(4) $$\Pi_{vdW}=-\frac{A_{H}}{6\pi h_{0}^{3}}$$

where $\Pi_{vdW}$ is van der Waals disjoining pressure, *h*_0_ is film thickness, and *A_H_* is the Hamaker constant.

The Hamaker constant is a coefficient that relates the interactive van der Waals energy to the separation distance between two molecules, where the interactive force is pairwise and independent of the intervening medium. It depends on the properties of materials and includes the Keesom and Debye polar molecular forces and London dispersion forces [25]. The Hamaker constant can be both positive and negative [22]. When the forces are attractive, the Hamaker constant is negative. In the case of complex interactions, there is an approximate formula for the Hamaker constant calculation [26], which is shown as Equation (5).

(5) $$A_{123}\approx \left(\sqrt{A_{11}}-\sqrt{A_{33}}\right)\left(\sqrt{A_{22}}-\sqrt{A_{33}}\right)\;$$

where *A*_123_ is the resulting Hamaker constant and *A*_11_, *A*_22_, and *A*_33_ are the Hamaker constants for each of three interacting media.

**Figure 2:**
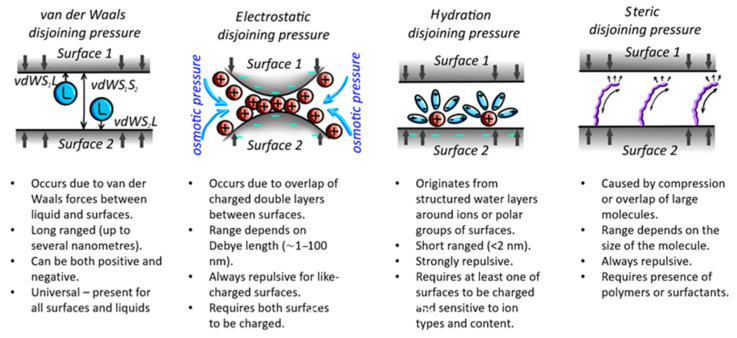
Main effects contributing to disjoining pressure in thin films.

In the case of the water, however, interactions between surfaces cannot be explained solely by van der Waals forces, since the disjoining pressure is also affected by electrostatic double-layer ionic osmotic pressure, structural forces arising from the hydration of water molecules on the surface, and steric pressure [25]. Figure 2 provides a schematic representation of these effects.

Electrostatic disjoining pressure (or double-layer osmotic pressure) is always positive due to the nature of the phenomenon. It occurs because of the formation of the electric double layer around the surfaces. Charged surfaces attract counterions and repel co-ions. When two charged surfaces come close to each other in the presence of electrolytes, overlap of the layers occurs, leading to increased osmotic pressure between the two surfaces due to the increased concentration of ions. With rubber, however, this pressure cannot occur, since rubber is not charged [27].

Hydration disjoining pressure occurs due to hydrogen bonds or polar interactions between the surface and the water, creating a hydration shell around the surface through interactions between the surface and clusters of water molecules surrounding ions. Despite the fact that hydration forces are critical in the lubrication of biosystems [28], rubber-like materials are less affected, since the rubber surface is not charged. Hydration forces are short-range (typically less than 3 nm) and are always repulsive, so they reduce adhesion. In the case of regular mineral aggregates, hydration forces can be stronger than van der Waals forces for liquids with high concentrations of multivalent cations such as Mg^2+^ and Ca^2+^, which are more hydrated [29]. Hydration shell formation becomes critical in the case of high surface area minerals, such as clay [30]. Concrete and cement can also develop stronger hydration shell around them.

Steric pressure is also repulsive, reducing adhesion due to the entropic penalty and osmotic pressure generated when layers of large molecules on opposing surfaces are compressed and forced to overlap. Hence, it only occurs in the presence of polymers or surfactants.

Beyond these main contributions, there are additional, more system-specific components of disjoining pressure that become significant under certain conditions. One such example is the structural (or solvation) disjoining pressure, which emerges when confined liquids develop oscillatory, layered ordering at molecular scales, commonly observed in ionic liquids or organised nanoparticle suspensions [31]. Other types of disjoining pressure, however, are more specific to the liquid medium and surfaces [32].

### 1.4. Effect of Adhesion on Friction

The practical role of adhesion in friction remains a matter of debate. Some suggest that, due to contamination, presence of air, and insufficient loading, especially in wet conditions, it is impossible for molecular interactions between surfaces to occur [33]. For the contact between two different surfaces to occur, the distance between surfaces should be within the range of van der Waals forces, which are usually equal to 10 nm. Thus, for adhesion forces to take effect, the distance between the surfaces should reach that limit [34].

Experimental studies show high influence of adhesion during the contact of rubber-like surfaces. In the studies [35,36], the work of adhesion was calculated for different rubber compounds, showing that the influence of adhesion on rubber compound is substantial, but can be reduced due to contamination. In the study [37], the adhesive contribution to the vehicle braking on a road was evaluated, results show that it depends on the sliding speed, reaching its maximum at lower speeds (0.1 cm/s to 1 cm/s). This study, however, did not consider any adhesive contribution for wet friction. A similar study by Al-Assi and Kassem [38] demonstrated that the adhesive bond energy between rubber and pavement materials significantly influences dry friction, with the adhesion component becoming more dominant at lower sliding speeds.

The study by Yib and Meyer [39] evaluates the adhesive contribution to friction by using the water with soap as a lubricant during the friction tests, stating that adhesive contribution to friction can reach 10%, decreasing with an increase in the sliding speed. The authors also conclude that surface texture enhances the influence of adhesion, probably due to increased pressure at the contact point, which allows stronger molecular binding.

The study by Bazlamit and Reza [23] evaluates the influence of the adhesion on runway friction by comparing British pendulum test results with water and liquid hand soap as a lubricant, assuming that liquid hand soap reduces the influence of adhesion to zero. Tests show a substantial (up to 30%) influence of adhesion on friction. This value, however, cannot be used as a reference, since liquid hand soap has high viscosity and also reduces the hysteresis friction as well by providing a thicker lubrication film. Moreover, it is clear that adhesion cannot be completely ignored, even with the hand soap as a lubricant.

It is clear that wet friction is susceptible to changes in liquid wetting capabilities. In the study [40], influence of the wettability, or contact angle, on surface friction is attributed to the reduction in adhesion interactions between two solids. In this case, the influence of the wettability of liquid on friction is explained by the introduction of a linear relationship between the friction coefficient and the work of adhesion during the interaction of a silica surface and a ball in the presence of liquid (Equation (6)).

(6) $$COF=k\frac{\partial A}{\partial L}S+\mu \;$$

where *COF* is coefficient of friction, *S* is the spreading pressure (Equation (3)), *A* is the contact area, *L* is the external load, *k* is the constant related to adhesive friction, and *μ* is a constant representing load dependence.

Similar dependence should also hold for runway friction, although the mechanisms of friction loss are different from those of the silica surface. Since one of the main sources of runway contamination and friction reduction is rubber build-up, it is important to understand how friction changes under different conditions. The presence of contamination and variations in water temperature affect actual friction and measurement results differently, causing possible unreliability of the measurements and risks for safe landings.

The goal of this study is to quantify the influence of adhesion on runway friction by using real rubber-contaminated pavement and models of rubber-contaminated pavement consisting of rubber plates with different textures and surface areas. Results of this study will help to understand the mechanisms of friction reduction for rubber-contaminated pavements and to improve the interpretation of friction testing results on such pavements.

## 2. Materials and Methods

### 2.1. Evaluation of the Effect of Adhesion on Friction

To assess the effect of adhesion on runway friction, measurements were performed using the British pendulum tester, on rubber-contaminated pavement surfaces, rubber (with different microtexture roughness), asphalt concrete, and cement concrete surfaces, using water with different surfactant content as a lubricant. Sodium lauryl sulphate, a common surfactant in dishwashing liquids, was used to reduce the contact angle of the liquid, thereby enhancing its wetting properties on the tested surfaces. The interfacial energy was monitored via static contact angle measurements on the rubber surfaces. For each series, friction tests were repeated on the same surface while gradually increasing the surfactant content. Before each test, the surface and pendulum slider were rinsed with the surfactant solution, and between measurement series, the slider was thoroughly cleaned with hot water to remove any residual surfactant.

Although surfactants may also affect film thickness, lubrication regime, and interfacial interactions, reduction in wettability is expected to be the primary mechanism contributing to friction reduction. This experimental design has been used previously in other studies and is supported by theoretical analyses [40,41,42]. Furthermore, despite the lack of precise theoretical quantification of the adhesive contribution, the proposed approach provides a practical and sufficiently simple method suitable for application under realistic runway pavement and testing conditions.

Additional tests were conducted using a roughened rubber plate at different temperatures, without surfactant, to assess the influence of temperature on friction. The rubber plate and the British pendulum tester slider were immersed in a liquid at the target temperature and conditioned for 30 min prior to testing. Contact angle measurements were performed at the same time to assess the free surface energy between the rubber plate and the water film.

### 2.2. Friction Measurements

**Figure 3:**
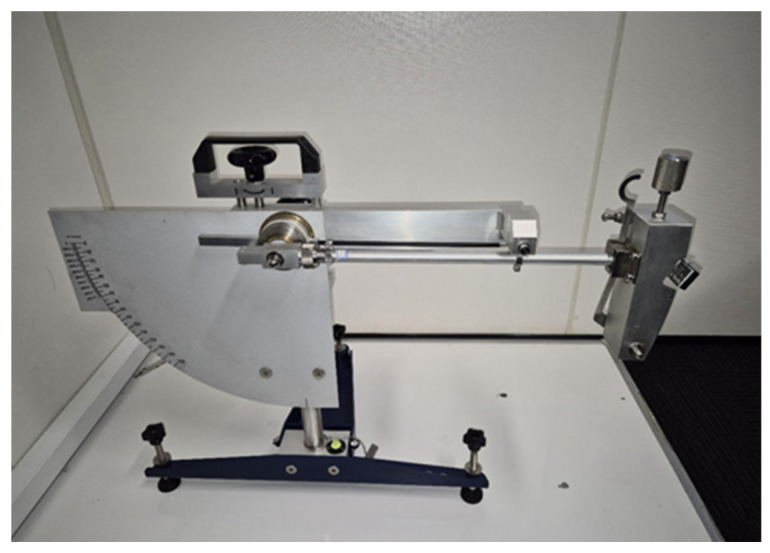
British pendulum tester.

As stated above, friction measurements were performed using a British pendulum tester (Figure 3). This method is widely used for measuring the friction of different surfaces due to its reliability and simplicity [43]. It is based on the measurement of the pendulum energy after sliding across a wet surface with a rubber slider attached to the end of the pendulum. The British Pendulum Number (BPN) is displayed on the plate behind the pendulum, reflecting the angle of the pendulum swing after sliding. During a test, five individual measurements were taken and subsequently averaged.

Although British Pendulum measurements do not directly replicate real aircraft braking conditions, this method allows accurate and repeatable measurements at the exact same location. Additionally, it has been widely used in the literature for runway surface assessment and has been shown to correlate well with Continuous Friction Measuring Equipment (CFME) results [44,45].

### 2.3. Contact Angle Measurements

**Figure 4:**
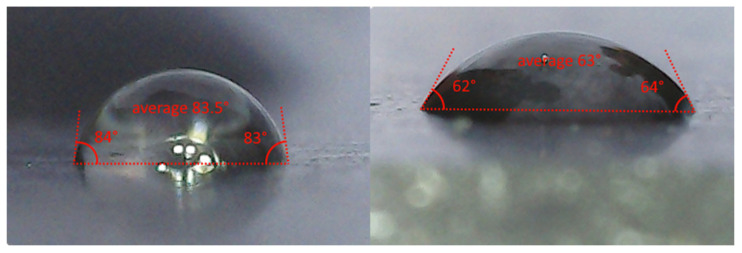
Contact angle measurement.

The sessile drop method is commonly used for the measurement of surface free energy between a liquid and a surface by means of measuring the contact angle between a droplet of liquid and a probe [46,47]. By comparing contact angles under different conditions, we can compare the surface free energy in each case—lower contact angles correspond to higher surface free energy. This information can then be used to estimate the dynamics of spreading pressure for liquids with different surfactant contents, since spreading pressure varies linearly with the cosine of the contact angle [25]. Figure 4 shows the process of contact angle measurement. All measurements were performed on a cleaned and degreased rubber plate, matching the material of the slider of the British pendulum tester. Each measurement was performed using a syringe and a digital microscope simultaneously with the friction measurement to avoid the influence of sample storage and temperature changes. The contact angle of liquid droplets was manually measured using AutoCAD 2022 (v 24.1).

Although manual contact angle measurement introduces uncertainty due to possible camera angle misalignment and droplet inconsistency, the measurement protocol included six individual measurements (three droplets per test), which allowed suitable precision to be achieved within the observed range of contact angles.

### 2.4. Effective Surface Area Measurements

Effective surface area measurements were performed using a digital microscope Inskam321 (Inskam, Huizhou, China). A thin slice of rubber was cut using a razor blade at approximately a 30° angle and the actual material edge length was measured. The estimated effective surface area coefficient was used to compare different rubber materials (Equation (7)).

(7) $$A_{e}=\frac{L_{measured}}{L_{edge}}$$

where *A_e_* is the estimated effective surface area coefficient, *L_measured_* is the measured length of the edge and *L_edge_* is the distance between opposite ends of the edge. The actual edge length was manually measured using AutoCAD 2022 (v 24.1).

**Figure 5:**
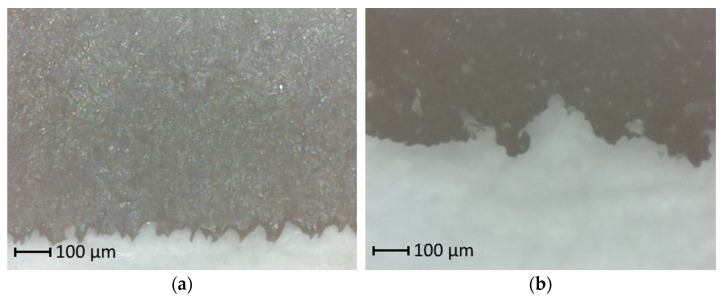
Surface area of rubber: (**a**) smooth rubber; (**b**) rough rubber.

Figure 5 shows the difference between the edges of smooth and sanded rubber cuts. As can be seen, the estimated effective surface area cannot be used to quantify the actual surface area of the specimen. However, this value can be used for comparing specimens.

### 2.5. Microtexture Measurements

**Figure 6:**
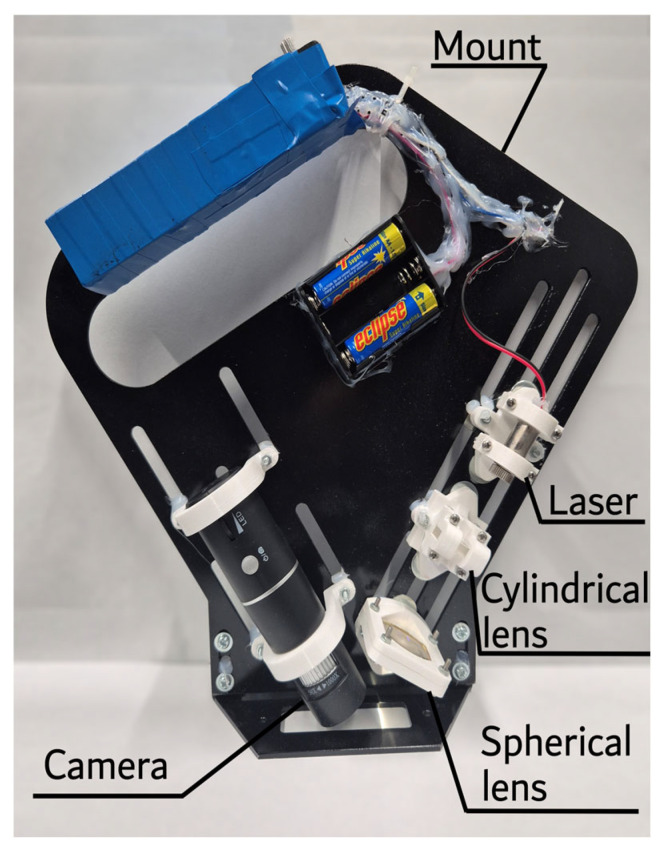
Laser profilometer for microtexture assessment.

Microtexture measurements were performed using a custom laser profilometer with a maximum vertical and horizontal resolution of 6 μm. The actual vertical resolution, however, is around 20 μm due to the thickness of the laser beam. The laser profilometer consists of a laser, a camera, and a set of lenses for the laser beam projection (Figure 6). One of the main features of this profilometer is its low cost and simplicity.

This profilometer setup was verified previously and showed high accuracy in the microtexture assessment [48]. The microtexture assessment algorithm consisted of the following steps: profile image processing, profile smoothing, macrotexture filtration using linear approximation, and microtexture profile assessment. This algorithm was verified previously, showing a strong correlation between microtexture and friction test results [49]. The average microtexture roughness (*R_a_*) was used to characterise the microtexture of the surface. Higher values of average microtexture roughness (*R_a_*) correspond to a rougher surface.

### 2.6. Testing Surfaces

**Figure 7:**
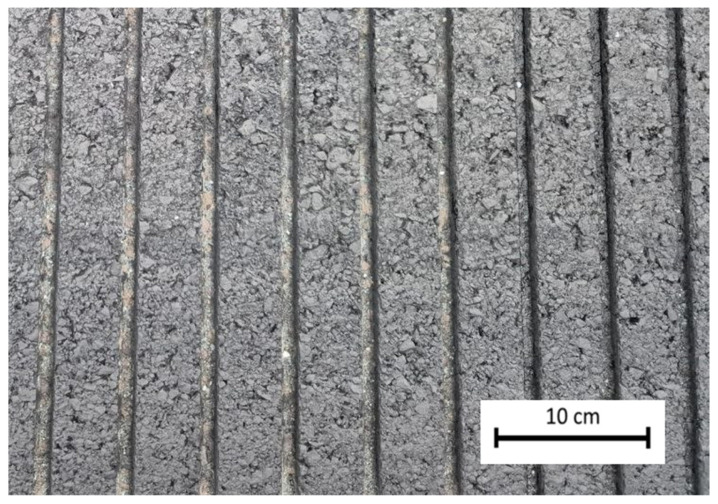
Rubber-contaminated pavement from the touchdown zone of the tested airport.

Tests were performed on rubber-contaminated pavement at a local Australian airport. The airport usually receives around 20 landings per day, predominantly by larger narrow-bodied passenger aircraft, such as the B737 and A321. Rubber contamination thickness was approximately 0.1 mm to 0.5 mm. The slider of the British pendulum tester was aligned between the grooves, so the friction results would not be affected by the irregularities. Figure 7 shows the rubber-contaminated runway pavement. Friction tests were performed at two random points on a touchdown zone with heavy rubber contamination. Although several testing points are required to ensure statistical robustness of the obtained data, this was not feasible due to operational limitations on the runway. Therefore, two points were selected as an illustrative example to demonstrate the concept.

**Figure 8:**
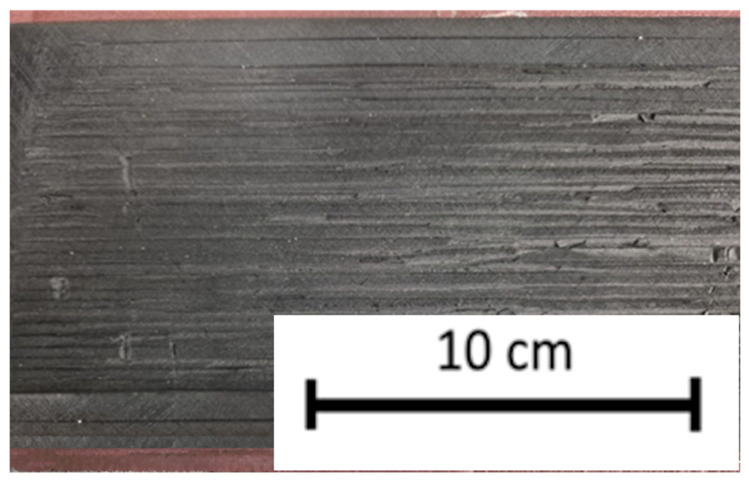
Sanded and roughened rubber plate to simulate a heavily contaminated runway surface.

As a reference, additional tests were performed on uncontaminated regular asphalt pavement, concrete slabs, and rubber plates with varying macrotextures. One of the rubber plates was progressively sanded and roughened in controlled stages to vary the surface microtexture and the effective contact area (Figure 8). At each stage, surface texture parameters and friction values were measured to assess the relationship between surface roughness, adhesive properties, and the resulting friction coefficient.

## 3. Results and Discussion

### 3.1. Viscous Pressure Calculation

To ensure the presence of adhesion in the contact area, the viscous pressure in the liquid film was approximated using the solution of Equation (2) under an assumption of exponential variation in film thickness in the contact area, as described by Moore [21]. Equation (8) for film thickness variation was used in the pressure approximation, as per Equation (8).

(8) $$h=0.001{\mathrm{e}}^{-1000x}$$

where *h* is the film thickness and *x* is the horizontal distance.

**Figure 9:**
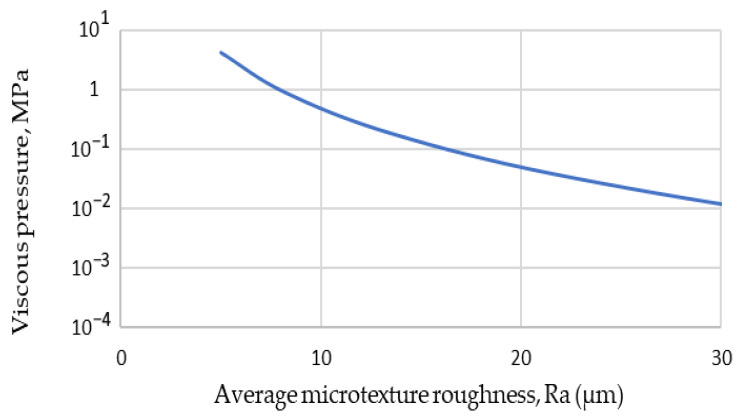
The viscous pressure and average microtexture roughness.

This form of film thickness variation was chosen based on the assumption of a 0.1 mm water film thickness in the inlet region and a pendulum arm swing radius of 0.5 m. With the speed of the pendulum arm of 2.84 m/s, the following dependence between viscous pressure and average microtexture roughness was obtained (Figure 9).

According to modelling studies [50,51], the average pressure in the contact zone of the British pendulum tester slider is around 0.5 MPa. It can be seen from Figure 9 that an average microtexture roughness above 10 μm is sufficient to avoid a significant influence of viscous hydroplaning on test readings. Similarly, in our previous study, we found a drastic reduction in BPN for surfaces with microtexture below 10 μm [48]. It does not imply complete hydroplaning of the slider on surfaces with lower microtexture, since Equation (2) is a two-dimensional form of the Reynolds equation. There will be a significant pressure reduction closer to the sides of the slider, and the pressure of the slider in the contact area is higher closer to the sides.

### 3.2. Effect of Surface Contact Angle on Friction

**Figure 10:**
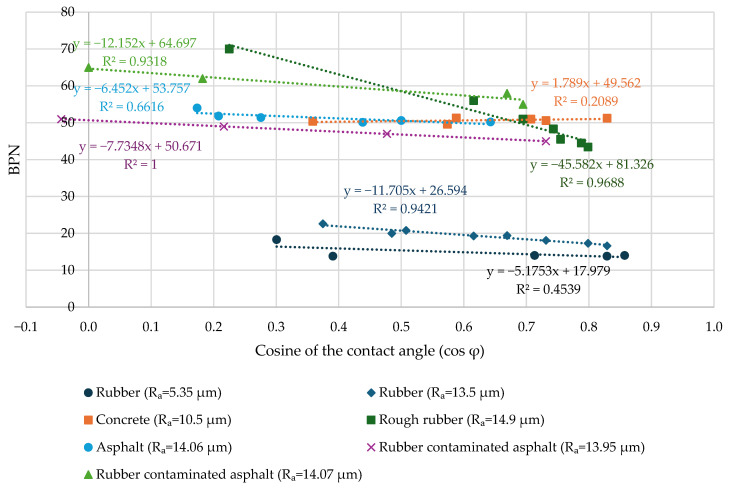
British Pendulum Number (BPN) for different materials and contact angles.

The results of the friction tests are shown in Figure 10. The results of the tests are additionally presented in Appendix A (Table A1). There is a significant reduction in the friction due to the reduction in the contact angle for rubber plates with higher microtexture and surface area, as well as for rubber-contaminated asphalt, and a moderate reduction in friction for smooth rubber and uncontaminated asphalt. The concrete sample did not experience friction reduction during the test. That means that adhesive contribution is significant for rubber plates and rubber-contaminated asphalt.

It should be noted that the dataset presented in this figure is exploratory in nature and limited in statistical robustness; therefore, the observed relationships should be interpreted as indicative trends rather than definitive quantitative correlations.

A linear fitting model was used to characterise the friction change, similarly to the methodology presented in [40]. Experimental results from other studies also support this approach [52,53]. We can assume that when the contact angle between the surface and the liquid drop reaches 0 and the cosine of the contact angle (cos φ) reaches 1 (complete wetting), no adhesion can occur. In that case, the full adhesive contribution for the tested materials is as follows (Table 1). The highest adhesive contribution was obtained for rough rubber, while the lowest was observed for cement concrete. Rubber-contaminated asphalt had 15.8% to 19.2% adhesive contribution, and uncontaminated asphalt had 12.4% adhesive contribution.

The dependence between the cosine of the contact angle and the BPN value is presented as Equation (9). This form is similar to Equation (6). Coefficient *a* in this case describes the influence of adhesion on friction, and coefficient *b* describes the influence of hysteresis on friction. Due to the limited number of data points and the narrow range of contact angles, a linear form of Equation (9) was used, similarly to that presented in previous studies [40].

(9) BPN=a×cos φ+b

where BPN is the British pendulum number, cos *ϕ* is the cosine of the contact angle between the liquid drop and a surface, and *a* and *b* are the slope and interception coefficients of the dependence.

### 3.3. Influence of Texture Parameters on Friction

The evaluation of the estimated effective surface area coefficient (Equation (7)) shows that it is 1.63 for smooth rubber, 1.86 for rough rubber, and 1.61 for rubber-contaminated asphalt. These results indicate that rubber-contaminated asphalt has an effective surface area similar to that of smooth rubber, which reduces the contribution of adhesion to friction compared with rough rubber.

**Figure 11:**
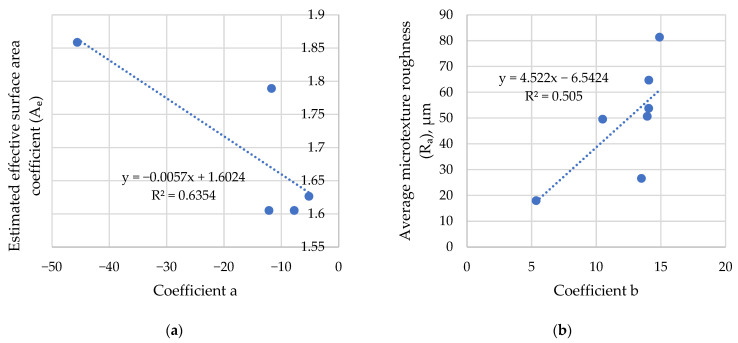
Influence of surface parameters on the dependence of friction on the cosine of the contact angle: (**a**) coefficient *a*; (**b**) coefficient *b*.

Figure 11 presents the influence of estimated surface area and average microtexture roughness on the coefficients *a* and *b* of Equation (9). There is a correlation between the estimated effective surface area and a coefficient characterising the influence of adhesion on the BPN value. This indicates that an increased surface area enhances the adhesive component of friction. Coefficient *b* correlates well with the microtexture, which indicates the influence of the average microtexture roughness on hysteresis friction, although it should be used carefully when comparing friction properties of different materials, such as a rubber plate and concrete.

### 3.4. Evaluation of Material Properties Effect

The influence of adhesion on friction depends on the material as well, since cement concrete is the least affected by adhesion. To evaluate the van der Waals disjoining pressure, the Hamaker constant A_123_ was calculated for all materials according to Equation (5) using Hamaker constants for each material from experimental studies. The Hamaker constant A_33_ for undiluted water is equal to 4.62 × 10^−20^ J according to [54]. In order to estimate the maximum disjoining pressure, we use a 0.3 nm distance as an approximate threshold at which adhesion occurs. This value is used in the literature for macroscopic surfaces [55].

Table 2 shows Hamaker constants for all tested materials, approximated Hamaker constants for contact interaction (Equation (5)) and van der Waals disjoining pressure (Equation (4)). It should be noted that the theoretical estimations based on Hamaker constants and disjoining pressure are intended to provide conceptual insight into the underlying interfacial mechanisms rather than serve as direct validation of the experimental findings. These calculations are used to support qualitative interpretation of the observed trends, while quantitative agreement between theory and experiment is beyond the scope of the present study.

All contact pairs experience negative van der Waals disjoining pressure, which means all materials are attracted to each other, with the highest attraction in the case of cement. Adhesive contribution for cement, however, is the lowest. This can be explained by hydration disjoining pressure, which can be significantly higher than van der Waals disjoining pressure in the case of such materials [59,60]. In the case of bitumen and rubber, however, disjoining pressure is predominantly governed by van der Waals forces since neither of those materials is significantly affected by other interactions. Hence, the higher adhesive contribution in the case of rubber can be explained by the approximated Hamaker constant difference.

Further reduction in contact angle leads to a reduction in the approximated Hamaker constant in all cases, which increases the disjoining pressure [61]. At some point, van der Waals disjoining pressure can become strongly repulsive, which further reduces the adhesive contribution [22]. At the same time, the presence of surfactants also increases the total disjoining pressure due to the steric effect after surfactant adsorption on the surface [62].

### 3.5. Effect of Surface Temperature on Friction

**Figure 12:**
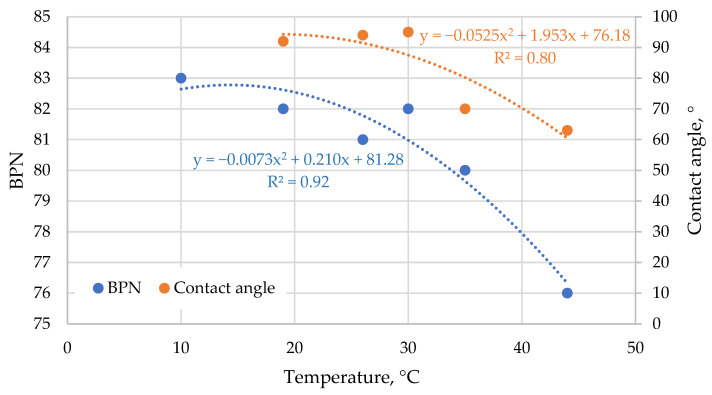
Influence of the surface and water temperature on the cosine of the contact angle and friction.

To confirm the adhesive nature of the temperature influence on friction results, similar tests were performed with no surfactant at different temperatures of the water, testing surface and a slider. A temperature range of 10 °C to 45 °C was used due to laboratory conditioning limitations and its relevance to the typical temperature range encountered on runways. Results of the test are shown in Figure 12. Due to the narrow range of contact angles, where cos *ϕ* varies approximately linearly with *ϕ*, and to improve visual clarity, the contact angle itself was used instead of its cosine.

The results show a simultaneous reduction in both contact angle and friction at higher temperatures, confirming the adhesive nature of the temperature influence on friction behaviour. This effect is less pronounced at ambient and sub-ambient temperatures. Contact angle measurements below 20 °C were complicated by the immediate formation of condensed water on the rubber surface due to the high dew-point conditions during testing. The same phenomenon may also influence friction measurements, as surface moisture alters the effective wetting regime.

To minimise this issue, tests would need to be conducted under controlled ambient temperatures, which is difficult to achieve in standard laboratory settings. It is also recommended that these tests be repeated using different rubber compounds representative of those used during aircraft landings and in friction-testing devices, as variations in rubber formulation may lead to different surface energy characteristics and, consequently, different temperature-dependent adhesive behaviour [63].

The obtained results explain the influence of weather conditions on friction measurement on runways [64]. Since binder- and rubber-covered materials are the main materials used in runways, friction is highly affected by the contact angle of the liquid, which can be reduced by temperature [65]. Adhesive friction can also be reduced by the presence of other contaminants, such as fine dust and pollutants that are known to reduce runway friction [66]. On the other hand, special surface treatments to increase the surface area of the rubber-contaminated zones or the hydrophobicity of rubber can significantly increase friction due to the adhesion effect.

## 4. Conclusions

This study demonstrates that adhesive forces make a significant contribution to runway friction, particularly in the presence of rubber contamination. Using British Pendulum Tester measurements, it was found that the approximate adhesive contribution ranged from negligible on cement concrete to more than 49% on rough rubber, with rubber-contaminated asphalt showing intermediate values of 16% to 19%. The relationship between friction and the cosine of the contact angle followed a linear trend, confirming that wetting properties directly affect friction, and the significant influence of adhesion on the runway friction. Results also revealed that effective surface area and microtexture play an important role in amplifying adhesive contributions, while materials such as cement exhibit low adhesion. Furthermore, evaluation of the Hamaker constants conceptually showed that higher values correlate with stronger adhesive contributions, which is consistent with the observed material-specific behaviour. Importantly, theoretical calculations of viscous pressure in the contact zone demonstrate that adhesive interactions are physically feasible, as contact between the water film and the surface is sustained by the predicted viscous pressure levels. Environmental factors such as water temperature and surface contamination further modify wetting behaviour, and therefore adhesion, providing a mechanistic explanation for the variability observed in continuous friction-measurement results under different weather conditions. This is further supported by controlled experiments conducted at different water temperatures. Overall, it was concluded that adhesion cannot be neglected when interpreting runway friction test data, particularly on rubber-contaminated pavements. Since the influence of such mechanisms has not been previously explored in an explanatory manner in the literature, the presented results, despite the limited statistical robustness of the dataset, provide conceptual insight that may support future improvements in the reliability of friction measurements and the design of surface treatments aimed at enhancing runway safety.

## Figures and Tables

**Table 1:** Adhesive contribution to friction for tested surfaces.

Material	Approximate Adhesive Contribution to BPN, %
Rubber (R_a_ = 5.35 µm)	30.0
Rubber (R_a_ = 13.5 µm)	34.1
Concrete (R_a_ = 10.5 µm)	0.0
Rough rubber (R_a_ = 14.9 µm)	48.9
Asphalt (R_a_ = 14.06 µm)	12.4
Rubber-contaminated asphalt (R_a_ = 13.95 µm)	15.8
Rubber-contaminated asphalt (R_a_ = 14.07 µm)	19.2

**Table 2:** Hamaker constants and van der Waals disjoining pressure for testing materials.

Material	Rubber	Bitumen	Cement
Hamaker constant, *A*_11_ (10^−20^ J)	5.7	5.2	16
Approximated Hamaker constant for rubber/water/surface contact, *A*_123_ (10^−20^ J)	0.057	0.031	0.441
van der Waals disjoining pressure, *Π_vdW_* (MPa)	−11.13	−6.12	−86.55
Reference	[56]	[57]	[58]

## Data Availability

The original contributions presented in this study are included in the article. Further inquiries can be directed to the corresponding author.

## Abbreviations

The following abbreviations are used in this manuscript:

CFME	Continuous friction measurement equipment
ICAO	International Civil Aviation Organization
BPN	British pendulum number

## Appendix A

**Table A1:** Friction and contact angle measurement results for different materials.

Material	Contact Angle, °	BPN
Rubber (R_a_ = 13.5 µm)	68	22.6
	61	20
	59.5	20.8
	52	19.3
	48	19.4
	43	18.1
	37	17.3
	34	16.6
Rubber (R_a_ = 5.35 µm)	72.5	18.3
	67	13.8
	44.5	14
	34	13.8
	31	14
Concrete (R_a_ = 10.5 µm)	69	50.4
	55	49.6
	54	51.3
	45	51
	43	50.6
	34	51.25
Rough rubber (R_a_ = 14.9 µm)	77	70
	52	56
	46	51
	42	48.3
	41	45.5
	38	44.5
	37	43.4
Asphalt (R_a_ = 14.06 µm)	80	54
	78	51.8
	74	51.4
	64	50.1
	60	50.6
	50	50.2
Rubber contaminated asphalt (R_a_ = 13.95 µm)	92.5	51
	77.5	49
	61.5	47
	43	45
Rubber contaminated asphalt (R_a_ = 14.07 µm)	90	65
	79.5	62
	48	58
	46	55
